# Reframing climate disasters through the lens of a climate necropolitics of health

**DOI:** 10.1093/inthealth/ihaf157

**Published:** 2025-12-30

**Authors:** Anushka Ataullahjan, Lesley Gittings

**Affiliations:** School of Health Studies, Faculty of Health Sciences, Western University, London, ON, Canada N6A 2K5; School of Health Studies, Faculty of Health Sciences, Western University, London, ON, Canada N6A 2K5

## Abstract

A climate necropolitics of health offers a critical framework that illuminates how climate change disproportionately harms marginalized populations by highlighting how the lives of certain individuals are protected and whose lives are expendable. We demonstrate how entrenched gender inequity, colonial legacies and systemic underinvestment in health and education have had negative health impacts for women and girls in the Swat Valley in Pakistan. We propose that a climate necropolitics of health asks us to move beyond seeing climate disasters as an isolated event, instead locating them in broader histories of environmental injustice, structural violence and social exclusion.

The 2022 floods in Pakistan submerged more than one-third of the country, with severe impacts on the health of women and girls. Findings from our recent participatory community-based study in the Swat Valley overwhelmingly demonstrated that the structural neglect of the health and well-being of women in the Swat Valley created precarity that was heightened when the floods hit.^1^ The 2022 floods worsened the existing limited access to health services. In turn, structural neglect caused women to go without access to sexual and reproductive health services, adequate nutrition and maternal health services. Infrastructural destruction also intensified women’s labour according to gendered allocation of household tasks. Opportunities to rebuild their lives were constrained for women due to a constellation of factors such as limited income-generating opportunities, entrenched *purdah* (gender segregation) and a lack of education.^1^

Necropolitics, a theory that describes how systems of power shape who lives and who dies, allows us to unpack the circumstances that gave rise to these experiences. For instance, we ask you to consider, what was the real cause of the impacts and disruptions to the health of the women described above? Was it decades of underinvestment in girl’s education with non-existent or non-functional girls’ schools because of entrenched patriarchal norms? Was it the complex geopolitical history, including a violent occupation of Swat by the Tehrik-i-Taliban Pakistan? Was it ethnic discrimination embedded in national resource allocation? Or was it entrenched poverty and caste-based social exclusion? The answers to these questions help us define the problem and in turn will shape how we intervene. We suggest that to confine our inquiry to the floods themselves is to obscure the entangled systems of chronic underinvestment, structural marginalization and environmental injustice that produced the conditions under which women disproportionately experienced the health consequences of the floods.

In this comment, we propose a climate necropolitics of health to support our inquiry, arguing that a climate necropolitics of health provides a lens through which to interrogate the broader sociocultural and sociopolitical structures within which health impacts of climate change and extreme weather events are situated and, in doing so, deepens our understanding of equity.

Necropolitics, developed by Achille Mbembe (2003), suggests that various destructive weapons of power are deployed to regulate who lives and who dies.^2^ Necropolitics also dictates the use of power to subject individuals to the status of ‘living dead’, illuminating divisions between social segments. Mbembe’s necropolitics builds on Foucault’s theory of biopower^3^—mechanisms of power that are focused on the regulation of human life. Mbembe also draws on the work of Fanon^4^ to argue that colonized geographies are spaces where violence is permitted.

In his work, Mbembe acknowledges the interrelationship between the extraction of natural resources with the war machine, speaking to the role of ecological violence in maintaining power structures.^5^ We propose extending this inquiry and highlight the importance of considering a ‘climate necropolitic of health’—a framing that highlights the ways in which climate violence is legitimized.^6^ This nascent concept of climate necropolitics allows us to highlight the interrelationship between human and environmental death and destruction. Furthermore, this theoretical framing brings into conversation a larger body of literature on climate coloniality, which contends that climate change represents a ‘colonisation of the atmosphere’ as a form of environmental racism.^7^ Indeed, it has been suggested that the disproportionate health issues borne by racialized peoples are an issue of structural violence and racial injustice.^8^

The framework of climate necropolitics allows us to trace the impacts of climate change on health. Climate change–related health impacts result from direct exposures to environmental effects of climate change, such as heat stress and exhaustion, and physical harm due to extreme weather events such as flooding and hurricanes, and indirect exposures such as allergens from changing seasonal patterns; food-, water- and vector-borne diseases; food insecurity resulting in mal- and undernutrition and mental health conditions such as eco-distress.^8^ While attention to the direct and indirect effects of health need to be examined and taken seriously, so too should the social and structural factors that create these environmental conditions in the first place. These risks include deaths and injuries, and are distributed unevenly, exacerbating underlying inequities and also creating new inequities.^9^ Certain population groups, based on age (e.g. children and the elderly), people living in poverty and isolation, racialized populations (including Black, Indigenous and other racialized peoples) and women and girls, carry a disproportionate burden of impacts due to a range of structural, social and physical vulnerabilities.^9^ A recent study found that racially minoritized groups, migrants and Indigenous communities across many different contexts experience a disproportionate burden of illness and mortality, and this is especially true in the Global South.^8^ Moreover, the social and economic effects of climate change are disproportionately borne by those who have benefited least from creating the conditions for climate change in the first place, with Global North countries responsible for 92% of greenhouse gas emissions.^10^

A climate necropolitics of health centres the health and well-being of those most marginalized. It forces us to interrogate our institutions that continue to be shaped by structural inequities related to gender, race, caste and class. It is a call to dismantle the existing systems of power such as colonialism, geopolitics (and the associated machines of war), patriarchy and racism that render some individuals more susceptible to the health impacts of climate change. Together, the suggestion is that preparing for and reducing the burden of climate-related morbidity and mortality is an issue of equity^9^ that requires attention to social and structural conditions. Climate change has become a reality that we can no longer deny, as we work towards creating strategies that mitigate its impacts, we must ensure that equity is at the centre of these approaches. If we value the lives of global citizens equally, such as flood-affected girls and women in Swat Valley, we must ensure that we are not re-creating existing inequities in climate change mitigation strategies.

## Data Availability

All data are included in the article.
